# Swine gut microbiome associated with non-digestible carbohydrate utilization

**DOI:** 10.3389/fvets.2023.1231072

**Published:** 2023-07-18

**Authors:** Sriniwas Pandey, Eun Sol Kim, Jin Ho Cho, Minho Song, Hyunok Doo, Sheena Kim, Gi Beom Keum, Jinok Kwak, Sumin Ryu, Yejin Choi, Juyoun Kang, Jeong Jae Lee, Hyeun Bum Kim

**Affiliations:** ^1^Department of Animal Resources Science, Dankook University, Cheonan, Republic of Korea; ^2^Division of Food and Animal Science, Chungbuk National University, Cheongju, Republic of Korea; ^3^Division of Animal and Dairy Science, Chungnam National University, Daejeon, Republic of Korea; ^4^Institute of Agricultural Science and Technology, Kyungpook National University, Daegu, Republic of Korea

**Keywords:** pigs, non-digestible carbohydrates, bacteria, fermentation, nutrition

## Abstract

Non-digestible carbohydrates are an unavoidable component in a pig’s diet, as all plant-based feeds contain different kinds of non-digestible carbohydrates. The major types of non-digestible carbohydrates include non-starch polysaccharides (such as cellulose, pectin, and hemicellulose), resistant starch, and non-digestible oligosaccharides (such as fructo-oligosaccharide and xylo-oligosaccharide). Non-digestible carbohydrates play a significant role in balancing the gut microbial ecology and overall health of the swine by promoting the production of short chain fatty acids. Although non-digestible carbohydrates are rich in energy, swine cannot extract this energy on their own due to the absence of enzymes required for their degradation. Instead, they rely on gut microbes to utilize these carbohydrates for energy production. Despite the importance of non-digestible carbohydrate degradation, limited studies have been conducted on the swine gut microbes involved in this process. While next-generation high-throughput sequencing has aided in understanding the microbial compositions of the swine gut, specific information regarding the bacteria involved in non-digestible carbohydrate degradation remains limited. Therefore, it is crucial to investigate and comprehend the bacteria responsible for the breakdown of non-digestible carbohydrates in the gut. In this mini review, we have discussed the major bacteria involved in the fermentation of different types of non-digestible carbohydrates in the large intestine of swine, shedding light on their potential roles and contributions to swine nutrition and health.

## 1. Introduction

Dietary carbohydrates (DCs) are principal substrates for maintaining physiological health and serve as an energy source for animals. In the diets of pigs, carbohydrates contribute to the majority of feed energy, accounting for approximately 60–70% of overall energy intake. In addition, the digestion of carbohydrates has significant impacts on various aspects of colonic functions, including the metabolism, balance of commensal flora and the health of large intestine epithelial cells (1).

DCs encompass a group of chemical substances and can be classified based on molecular sizes, ranging from simple mono- and disaccharides to complex compounds with intricate structures (2). Moreover, carbohydrates can be divided into two nutritional categories based on chemical classification. The first category is digestible carbohydrates (DGCs), which are metabolized by the host’s endogenous enzymes and absorbed in the small intestine. This category includes monosaccharides, disaccharides, and polysaccharides such as starches. The second category is dietary fiber, which consists of non-digestible soluble and insoluble carbohydrates and lignin. These components have the potential to be degraded through microbial fermentation in the large intestine (3). Non-starch polysaccharides (NSPs) are a component of dietary fiber. American Association of Cereal Chemists (AACC) defined the dietary fiber in 2000 as edible plant parts or comparable carbohydrates that are resistant to digestion and absorption in the small intestine but can be completely or partially fermented in the large intestine (4). Dietary fiber contains a substantial amount of energy, but the majority of enzymes required for its breakdown are not encoded in the mammalian genome.

Starch is the principal source of energy for monogastric animals and cereals are the primary source of starch in animal feeds (5). Starch is a polysaccharide composed of polymers amylose and amylopectin. Resistant starch (RS), on the other hand, refers to starches that resist digestion in the small intestine by amylases and instead reach the large intestine, where they are available for bacterial fermentation (6).

NSPs (cellulose, pectin, and hemicellulose), RS and non-digestible oligosaccharides (NDOs) (Fructo-oligosaccharide and Xylo-oligosaccharide) are the major types of non-digestible carbohydrates (NDCs). The large intestine serves as an anaerobic digestive environment for complex molecules, such as NDCs. It is predominantly colonized by obligate anaerobic microorganisms, although a small number of aerobic and facultative microorganisms are also present (7, 8). These microorganisms within the large intestine utilize NDCs as their primary energy sources. As a result, they produce Short Chain Fatty Acids (SCFAs), vitamins, and participate in various metabolic processes. Moreover, these microorganisms engage in intricate interactions with host cells and the host immune system (9–11).

With the aid of next-generation high-throughput sequencing, researchers have been able to comprehend the gut microbial composition of swine. However, knowledge concerning the gastrointestinal tract microbiome that facilitates the fermentation of NDCs remains limited, despite several research endeavors aimed at understanding such bacterial species. Therefore, this mini review aims to consolidate information regarding the major bacterial species involved in the fermentation of different types of NDCs in the large intestine of swine.

## 2. Degradation of NDCs by the swine gut microbiota

DGCs are such carbs that can be digested by host’s enzymatic system (3), whereas NDCs are those carbs that resist the action of salivary and intestinal digestive enzymes and hence are fermented by microbes in the large intestine (12).

The carbohydrates in the swine feed like cereal grains, legumes, oil seeds, and potato are also composed of DGCs and NDCs (13). The legumes and oil seeds are source of protein however the cell wall of these crops contains NDCs (14). NDCs have a significant role in pig diets, and it is necessary to add a certain amount to ensure proper physiological functioning (15).

**Figure 1:**
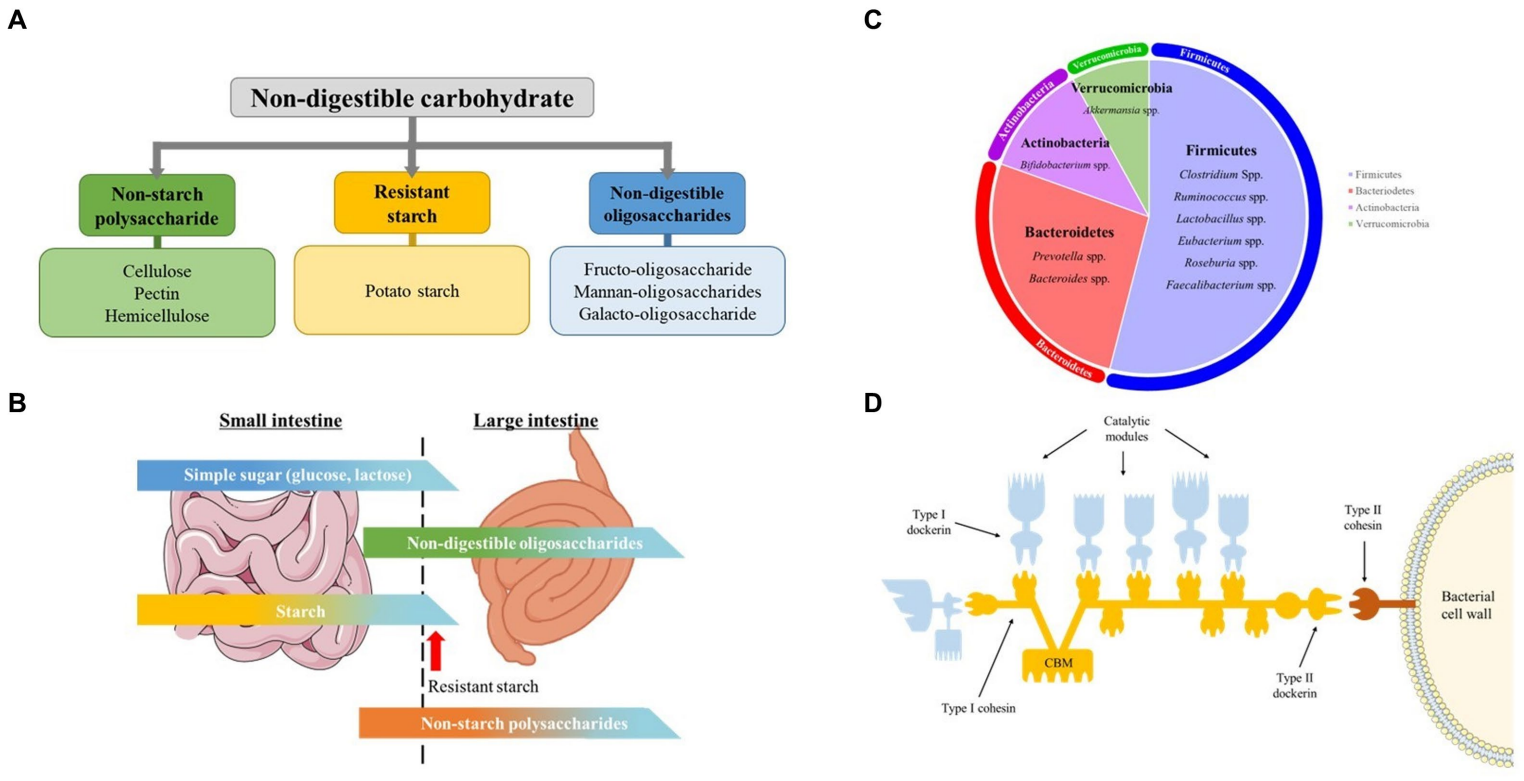
Non-digestible carbohydrates, pig gut microbiome, carbohydrate digestion in the intestine, and celluosome structure. **(A)** Classification and examples of non-digestible carbohydrate addressed in this mini review. **(B)** Schematic illustration of digestion of different carbohydrates in small and large Intestine. The figure gives a rough idea on the amount of carbohydrates digested in the small and large intestine. Modified from Bach Knudsen et al. **(C)** Predominant genus of swine gut microbiota. Each area of the circle signifies the domination of the respective phyla in the swine gut. **(D)** Cellulosome structure. The Type I dockerins, attached to the catalytic subunit (blue) interacts with the cohesin (yellow) of the primary CipA scaffoldin protein forming cellulosome complex. The cellulosome is attached to the bacterial surface through interaction of Type II dockerin in CipA with Type II cohesin module of a membrane-bound protein (red). The Cellulosome complex binds to cellulose through cellulose-binding module (CBM) of the CipA primary scaffoldin protein. Modified from Gilbert H.J.

As summarized in Figure 1A, NDCs are a distinct group of carbohydrates found almost entirely in plants and is generally classified as NSPs (cellulose, hemicellulose, pectin), RS (potato starch) and NDOs (Fructo-oligosaccharide, Xylan-oligosaccharide, soybean Oligosaccharide) (16).

NDCs are either water soluble or insoluble based on its solubility in water. Insoluble NDCs includes cellulose, hemicellulose, lignin, whereas soluble NDCs includes pectin, β-glucan, fructan, mucilage, gum, and psyllium fiber (17, 18). The most commonly present NDCs are cellulose, hemicellulose and pectic substances.

Digestion of simple carbs and starch occurs predominantly by enzymatic digestion, whereas the complex carbs that are resistant to host’s digestive enzymes are degraded by microbial fermentation after reaching large intestine (19).

Even though a host’s genome does not encode the enzymes required to break down the linkage between the monomers in NSPs and NDOs, 20 to 25% of NSPs and 40 to 95% of NDOs is found to be degraded while passing through the small intestine (Figure 1B). This breakdown is facilitated by the microbial enzymes of the microflora present in this part of the gut and not by the host’s enzymatic system (20). Nevertheless, the major types of carbs reach the large intestine, and those available for fermentation are plant cell wall polysaccharides, also known as NSPs, RS, and NDOs. Those carbohydrates are fermented by the swine gut microbiota.

Gastro-intestinal tract microbiota is defined as the ecological community made up of commensal, symbiotic and potentially pathogenic microorganisms that harbors the gut (17). The gut microbial composition of the swine is of great significance, as it affects the overall physiology and health, along with the feed conversion ratio. The swine gut microbiota are mainly made up of anaerobic & facultative anaerobic bacteria, and more than 90% of these bacteria belong to the phyla Firmicutes, Proteobacteria and Bacteroidetes (Figure 1C) (21–24). Several studies have shown ‘core’ genera consisting of *Prevotella, Clostridium, Ruminococcus, Lactobacillus, Faecalibacterium, Bacteroides, Fusobacterium,* and *Alloprevotella* in a larger portion of studied healthy pigs (25, 26). In this review, we will discuss major bacterial species involved in fermentation of different types of NDCs in the large intestine of swine.

### 2.1. Fermentation of NSPs by swine gut microbiota

NSPs comprise plant cell wall polysaccharides (Cellulose, hemicellulose, Pectin), structural non-polysaccharide (lignin) and non-structural polysaccharides. (7, 27). Numerous parameters, including the animal species, solubility, chemical composition, and consumption amount, influence the ease of digestion of NSPs. The order of microbial degradation in the large intestine is sugar residues = NDOs > Starch residues > Soluble NSP > RS=Insoluble NSP (3). Lignin, a component of plant cell wall, is however not digested by the enzymes of the small intestine and neither fermented by the gut bacteria. It is however supposed to impact the fermentability of other components in the diet (12, 28).

The degradation of complex fiber by the fibrolytic bacterial community is mainly carried out by several anaerobic gut microbes that possess the ability to produce enzymes. They belong to the dominant bacteria groups including *Bacteroides*, *Roseburia*, *Ruminococcus* or *Bifidobacterium* species (Table 1). Several specific as well as multi-carbohydrate degrading bacteria is found in swine gut. The anerobic bacteria mainly degrade cellulose through cell-bound organelle-like structure, cellulosome (46). Cellulosome is a large multi-enzyme complex bound to the bacterial cell wall, that helps degrade plant cell wall polysaccharides into usable sugars (47, 48). It basically consists of 2 major subunits: the enzymes and the noncatalytic subunit scaffoldin (Figure 1D). Scaffoldin possess 6 cohesin domains which bind with the dockerin module present in the enzymes and hence forms the functional cellulosomal-complex. Scaffoldin also has one another module, the cellulose-binding domain (CBM) that helps bind to the cellulosic substrates.

**Table 1:** Gut bacteria involved in fermentation of non-digestible carbohydrates.

Non-Digestible Carbohydrates (NDCs)		Genus	Species	References
NSP^1^	Cellulose	*Clostridium*	*leptum, herbivorans*	(29)
		*Ruminococcus*	*flaveciens, albus,*	(8, 30)
			*champanellensis*	(31, 32)
		*Fibrobacter*	*intestinalis, succinogenes*	(33)
		*Bacteroides*	*succinogenes*	(34)
	Hemicellulose	*Butyrivibrio*	*fibrisolvens*	(8, 35)
		*Bacteroides*	*ruminicola, xylanisolvens*	(36, 37)
		*Ruminococcus*	*champanellensis*	(31, 32)
	Pectin	*Bacteroides, Prevotella*	–	(38, 39)
Resistant Starch	–	*Ruminococcus*	*bromii*	(40, 41)
		*Bifidobacterium*	*adolescentis*	
		*Bacteroides*	*thetaiotaomicron*	
		*Eubacterium*	*rectale*	
NDO	FOS	*Bifidobacterium* *Lactobacillus*	*–* *plantarum, acidophilus*	(42, 43) (44, 45)
	GOS			
	MOS			
	SBOS			
	IMO			

NSP, Non-Starch Polysaccharide; NDO, Non-Digestible Oligosaccharide; FOS, Fructo-oligosaccharide; GOS, Galacto-oligosaccharide; MOS, Mannan-oligosaccharide; SBOS, Soybean-oligosaccharide; IMO, Isomalto-oligosaccharide.

*Ruminococcus flavefaciens* (*R. flavefaciens*), one of the predominant and important cellulolytic bacteria, degrades cellulose and a variety of plant cell walls (30) using a high-molecular-weight enzyme complex, cellulosome (49). It is essential for the *R. flavefaciens* to bind to the cellulose in order to break it down. Another bacteria, *Ruminococcus albus* (*R. albus*), is also a well-known specialist cellulose-degrading bacterium found in rumen and gastro-intestinal tract of herbivores, but it has also been isolated from swine gut (8). It is also known to produce a cellulosome-like complex. However, it is supposed to employ another mechanism for adhesion to cellulose. It has been found that a cellulose-binding protein belonging to the Pil-protein family is involved in attachment (50). *Ruminococcus champanellensis* (*R. champanellensis*), a recently identified cellulolytic strain from human feces, ferments cellulose and xylase, while metabolizing cellobiose to acetate, succinate, ethanol, dihydrogen and small quantities of formate and lactate (31, 32).

*Clostridium* (*C. leptum* and *C. herbivorans*), which are normal inhabitants of the pig’s intestine, also possess multi-enzyme system including cellulosome and xylanosome, which aid in the breakdown of complex cellulosic polymers and several cellulosic by-products (29). However, it should be noted that not all polysaccharidase activities in *R. flaveciens* or the cellulolytic *Clostrdia* are linked to a cellulosome (51).

*Bacteroides*, another highly prevalent genus in swine gut, possess a high concentration of the carbohydrate-active enzyme (CAZyme) genes. These enzymes enable *Bacteroides* to degrade various components of plant cell wall, like glucronylxylans, xyloglucans and pectin (52). Moreover, a unique feature of *Bacteroidetes* is the presence of polysaccharide utilization locus (PUL), which consists of linked genes involved in the saccharification of complex carbohydrates, such as glycans (53, 54). *Bacteroides ruminicola* (*B. ruminicola*) and *Bacteroides xylanisolvens* (*B. xylanisolvens*) are significantly involved in the degradation of xylan (36, 37). Another species, *Bacteroides succinogens* (*B. succinogens*), isolated from the swine’s large intestine, also possesses the ability to degrade cellulose (34). Metagenome-assembled genomes (MAGs) have identified several members of the Bacteroidaceae family, including *Bacteroides fragilis (B. fragilis)*, *Bacteroides heparinolyticus (B. heparinolyticus)*, *Bacteroides stercoris (B. stercoris)*, *Bacteroides thetaiotaomicron (B. thetaiotaomicron)*, *Bacteroides uniformis (B. uniformis)*, and *Bacteroides xylanisolvens (B. xylanisolvens)*. These bacteria have been found to possess the carbohydrate-active enzyme (CAZyme) genes involved in the degradation of starch, pectin, fucose oligosaccharides, rhamnose oligosaccharides, and other complex carbohydrates. Additionally, using metagenome-assembled genomes (MAGs), it has been predicted that *B. thetaiotaomicron* and *Bacteroides ovatus* (*B. ovatus*) possess PULs.

Several species of *Fibrobacter*, including *Fibrobacter intestinalis* (*F. intestinalis*) and *Fibrobacter succinogenes* (*F. succinogenes*), possess the ability to ferment NSPs. These species, found in the caeca of pigs, have gained significant attention due to their relatively higher fibrolytic activity (33). *F. succinogenes,* in particular, is known for its efficient degradation of cellulose. The specific mechanism by which it utilizes cellulose is still not fully understood, but it has been proposed that *F. succinogenes* binds to cellulose through a protein present in its outer membrane, facilitating the degradation of cellulose (55, 56). *Bacteroides*, along with *Prevotella*, is known to contain carbohydrate-active enzymes (CAZymes) and play a major role in the degradation of pectin, a component of plant cell walls. However, it is important to note that while these bacterial genera contribute to the breakdown of various dietary carbohydrates, cellulose degradation is mainly carried out by other cellulolytic bacteria such as *Ruminococcus, Fibrobacter*, and certain species of *Clostridium* (38, 39). It is also widely known that *Butyrivibrio* spp., found in swine colon, can hydrolyze hemicellulose (8). *Butyrivibrio fibrisolvens* is a ruminal hemicellulose- degrading bacteria but also show modest cellulolytic activity (35).

### 2.2. Fermentation of RS by swine gut microbiota

Starch is the principal source of energy for monogastric animals, and cereals are the primary source of starch in animal feeds (5). Starch is a polysaccharide composed of polymers: amylose and amylopectin. RS, on the other hand, refers to starches that escape digestion in small intestine by the amylases and reach the large intestine, where they become available for bacterial fermentation (6).

Based on their physiochemical characteristics, resistant starch (RS) is classified into five different types. RS1 refers to starches that are physically inaccessible as they are located inside a fiber-protein matrix and are resistant to breakdown even with normal cooking. RS2 is a type of starch found in green bananas and raw potatoes, which can be reduced by thermal treatment. RS3 refers to retrograded starches that occur when starchy foods like bread or potatoes are gelatinized through heating and then undergo retrogradation upon cooling. RS4 is a group of resistant starches that are generated through chemical modifications such as esterification, etherification, and cross-linking. RS5 is predominantly associated with amylose-lipid V-type complexes, such as starch-monoglycerides and starch-fatty acids (57, 58). Given that starchy ingredients consumed by pigs typically contain significant quantities of RS1, RS2, and RS3, these three types of resistant starch are commonly regarded as the primary ones in the swine industry.

Several types of gut bacteria are involved in fermenting resistant starch in the hindgut, which leads to the production of SCFAs (such as acetate, butyrate, propionate, and valerate), gasses (including CO₂, H₂, and CH₄), as well as lesser amounts of organic acids (like lactate, succinate, and formate), branched SCFAs, and alcohols (such as methanol and ethanol) (57).

The main three phyla involved in starch fermentation are Firmicutes, Bacteroidetes, and Actinobacteria, which collectively account for 95% of the total gut bacteria in mammals. Numerous studies have indicated the interactions between resistant starch and microorganisms in the gut. It has been observed that as the levels of RS increase, there is an increase in the populations of the *Bifidobacteria* and *Lactobacillus* genera. Specifically, *Lactobacillus sobrius* and *Lactobacillus amylovorus* have been identified as the major amylolytic genera in the digestive system of swine. It is important to note that not all *Bifidobacteria* species are involved in the degradation of RS. However, *Bifidobacterium breve, Bifidobacterium dentium*, and *Bifidobacterium pseudolongum* have shown extracellular starch-degrading activities (59). A human study also evaluated the roles of four dominant amylolytic bacteria in the human colon, namely *Bacteroides thetaiotaomicron (B. thetaiotaomicron), Ruminococcus bromii (R. bromii), Eubacterium rectale (E. rectale)*, and *Bifidobacterium adolescentis (B. adolescentis)*, in the breakdown and utilization of RS. The findings of the study indicated that *R. bromii* exhibited a much greater capacity for RS degradation compared to the other three bacteria. However, all four bacteria demonstrated the ability to utilize RS (40, 60).

The starch-utilization system of *B. thetaiotaomicron* has been thoroughly investigated and found to possess a starch-utilization-structure (sus) gene clusters, which play a role in binding and utilization of starch (41, 61). On the other hand, the remarkable starch-degrading capacity of *R. bromii* is believed to be attributed to cohesion (Coh)-dockerin interactions, which are particularly significant in cellulosomal enzyme systems (62).

*E. rectale* depends on a large extracellular amylase attached to its cell wall, along with some membrane-associated binding proteins and hydrolases to use resistant starch, however it is not a primary degrader (63, 64) (Table 1).

### 2.3. Fermentation of NDOs by swine gut microbiota

NDOs are a type of oligosaccharides that cannot be broken down by mammalian endogenous enzymes (65). The terms “resistant oligosaccharides,” “NDOs” and “resistant short chain carbohydrates (RSCC)” are interchangeable and refer to the same compound. NDOs, such as fructo-oligosaccharides (FOS), transgalacto-oligosaccharides (TOS), xylo-oligosaccharides (XOS), and soybean oligosaccharides, occur naturally in legume seeds and cereals. They can also be artificially synthesized. These examples represent some common types of NDOs. Numerous studies have demonstrated that the presence of NDOs leads to an increase in *Bifidobacterium* species compared to other bacteria (42, 43). While *Bifidobacterium* shows a strong preference for fermenting NDOs, other bacteria such as *Lactobacillus, Bacteroides,* and *Clostridium* also have the ability to ferment NDOs, albeit at lower levels.

Lactic acid bacteria, such as *Lactobacillus* species can utilize simple carbohydrates broken down by other bacteria (66). However, their ability to utilize complex carbohydrates is generally limited, with only certain species such as *Lactobacillus acidophilus* and *Lactobacillus plantarum* having the capability to utilize NDOs (44, 45).

*Bifidobacterium* species are known for their production of glycolytic enzymes, which enable them to efficiently utilize NDOs. These enzymes allow *Bifidobacterium* strains to hydrolyze various monosaccharides and glycosidic linkages, providing them with a broader range of carbohydrate substrates. In contrast, other enteric bacteria such as *Escherichia coli, Streptococcus*, and *Lactobacillus* generally exhibit less diversified enzyme activities and lower levels of activity compared to *Bifidobacterium* (67) (Table 1).

## 3. SCFAs from complex carbohydrate fermentation

NDCs play a crucial role in the overall health of pigs at all stages of life. When these carbohydrates undergo fermentation in the gut, they contribute to the production of SCFAs, gasses, and organic acids.

The anaerobic fermentation of complex carbohydrates in the large intestine primarily produces SCFAs, which are small organic monocarboxylic acids (68, 69). SCFAs have several beneficial effects on the host’s gut health. They contribute to the maintenance of intestinal barrier integrity, promoting a healthy gut lining and preventing the entry of harmful substances into the bloodstream. SCFAs also stimulate the production of mucus, which forms a protective layer in the gut and aids in the proper functioning of the digestive system. Furthermore, SCFAs have anti-inflammatory properties and help regulate the immune response in the gut, reducing the risk of inflammation-related conditions. Overall, the production of SCFAs through the fermentation of complex carbohydrates plays a crucial role in supporting gut health and protecting against intestinal inflammation (70). The major SCFAs produced in the gut are acetate, propionate, and butyrate (71). Among these, butyrate plays a crucial role as it serves as the primary energy source for colonocytes (72). Propionate, on the other hand, is transported to the liver where it contributes to gluconeogenesis. Acetate, being the most abundant SCFA, has various important functions including improving cholesterol metabolism and lipogenesis, regulating the immune system, and exhibiting anti-inflammatory responses (73–75). SCFAs cause a decrease in the pH of the large intestine, which helps inhibit the growth of pathogenic microorganisms and facilitates the absorption of calcium and magnesium (71). Furthermore, SCFAs can function as signaling molecules by binding to G protein-coupled receptors (GPCRs) present in gut epithelial cells and immune cells (76). In both anaerobic ecosystems and the alimentary canal, an inevitable byproduct of microbial fermentation is gas, with representative gasses being H₂ and CO₂.

## 4. Conclusion

In addition to the energy production through fermentation of NDCs, they also play a crucial role in maintaining gut health by producing a diverse range of metabolites, such as SCFAs. Gut microbes contribute to host health through SCFA production, lowering pH, and synthesizing vitamins.

The specific gut anaerobes involved in the breakdown of complex carbohydrates in the large intestine are still not fully understood. Advancing our knowledge in this area is crucial to better understand the microbial ecosystem and its impact on swine health. Prioritizing research efforts to identify the bacterial species responsible for fermenting various dietary fibers will not only contribute to maintaining overall health but also promote optimal growth and well-being of swine. This knowledge can aid in developing targeted interventions and strategies to optimize gut health and maximize the benefits of complex carbohydrate fermentation in swine. Identifying and understanding the functional roles of different bacterial species involved in the fermentation of dietary fibers can greatly enhance swine production. By optimizing diets and developing targeted interventions based on this knowledge, we can promote efficient fermentation and maximize the utilization of dietary fibers by beneficial gut bacteria. This can lead to improved nutrient absorption, enhanced gut health, and ultimately, better swine production outcomes. Additionally, such advancements can contribute to more sustainable and efficient farming practices by reducing feed waste and improving the overall utilization of available resources.

## Author contributions

JC, MS, and HK: conceptualization. SP, EK, HD, SK, GK, JiK, SR, YC, JuK, and JL: resources. HK: supervision. SP, EK, and HK: writing – original draft. JC, MS, and HK: writing – review & editing. All authors contributed to the article and approved the submitted version.

## Funding

This work was supported by National Research Foundation of Korea (the Ministry of Education: 2021R1I1A3059910 and the Ministry of Science & ICT: 2019M3A9F3065227).

## Conflict of interest

The authors declare that the research was conducted in the absence of any commercial or financial relationships that could be construed as a potential conflict of interest.

## Publisher’s note

All claims expressed in this article are solely those of the authors and do not necessarily represent those of their affiliated organizations, or those of the publisher, the editors and the reviewers. Any product that may be evaluated in this article, or claim that may be made by its manufacturer, is not guaranteed or endorsed by the publisher.

## Abbreviations

DCs: Dietary carbohydrates
DGCs: Digestible carbohydrates
NSPs: Non-starch polysaccharides
RS: Resistant starch
NDOs: Non-digestible oligosaccharides
NDCs: Non-digestible carbohydrates
SCFAs: Short Chain Fatty Acids.

## References

[1] Knudsen KEB Hedemann MS Laerke HN. The role of carbohydrates in intestinal health of pigs. Anim Feed Sci Tech. (2012) 173:41–53. doi: 10.1016/j.anifeedsci.2011.12.020

[2] Cummings JH Stephen AM. Carbohydrate terminology and classification. Eur J Clin Nutr. (2007) 61:S5–S18. doi: 10.1038/sj.ejcn.1602936 17992187

[3] Erik K Knudsen B Lærke HN Jørgensen H. Carbohydrates and carbohydrate utilization in swine In: Chiba LI, editor. Sustainable Swine Nutrition. Hoboken, NJ: John Wiley & Sons (2013). 109–37.

[4] Devries J Camire M Cho S Craig S Gordon D Jones JM. The definition of dietary fiber. Cereal food world (2001) 46:112–29.

[5] Stevnebo A Sahlstrom S Svihus B. Starch structure and degree of starch hydrolysis of small and large starch granules from barley varieties with varying amylose content. Anim Feed Sci Tech. (2006) 130:23–38. doi: 10.1016/j.anifeedsci.2006.01.015

[6] Walsh SK Lucey A Walter J Zannini E Arendt EK. Resistant starch-an accessible fiber ingredient acceptable to the Western palate. Compr Rev Food Sci F. (2022) 21:2930–55. doi: 10.1111/1541-4337.12955 35478262

[7] Chassard C Lacroix C. Carbohydrates and the human gut microbiota. Curr Opin Clin Nutr. (2013) 16:453–60. doi: 10.1097/MCO.0b013e3283619e63 23719143

[8] Varel VH Yen JT. Microbial perspective on fiber utilization by swine. J Anim Sci. (1997) 75:2715–22. doi: 10.2527/1997.75102715x, PMID: 9331875

[9] Rowland I Gibson G Heinken A Scott K Swann J Thiele I. Gut microbiota functions: metabolism of nutrients and other food components. Eur J Nutr. (2018) 57:1–24. doi: 10.1007/s00394-017-1445-8, PMID: 28393285 PMC5847071

[10] Flint HJ Duncan SH Scott KP Louis P. Interactions and competition within the microbial community of the human colon: links between diet and health. Environ Microbiol. (2007) 9:1101–11. doi: 10.1111/j.1462-2920.2007.01281.x, PMID: 17472627

[11] Hooper LV Wong MH Thelin A Hansson L Falk PG Gordon JI. Molecular analysis of commensal host-microbial relationships in the intestine. Science. (2001) 291:881–4. doi: 10.1126/science.291.5505.881, PMID: 11157169

[12] Chanmuang S Nguyen QA Kim HJ. Current research on the effects of non-digestible carbohydrates on metabolic disease. Appl Sci-Basel. (2022) 12:3768. doi: 10.3390/app12083768

[13] Navarro D Abelilla JJ Stein HH. Structures and characteristics of carbohydrates in diets fed to pigs: a review. J Anim Sci Biotechnol. (2019) 10:39. doi: 10.1186/s40104-019-0345-6 31049199 PMC6480914

[14] Knudsen KE. Fiber and nonstarch polysaccharide content and variation in common crops used in broiler diets. Poult Sci. (2014) 93:2380–93. doi: 10.3382/ps.2014-03902, PMID: 25012855

[15] Wenk C. The role of dietary fibre in the digestive physiology of the pig. Anim Feed Sci Tech. (2001) 90:21–33. doi: 10.1016/S0377-8401(01)00194-8

[16] Jha SK Singh HR Prakash P. Chapter 1 - dietary Fiber and human health: an introduction In: Samaan RA, editor. Dietary Fiber for the Prevention of Cardiovascular Disease. Cambridge, MA: Academic Press (2017). 1–22.

[17] Kalyani Nair K Kharb S Thompkinson DK. Inulin dietary Fiber with functional and health attributes—a review. Food Rev Intl. (2010) 26:189–203. doi: 10.1080/87559121003590664

[18] Mudgil D Barak S. Composition, properties and health benefits of indigestible carbohydrate polymers as dietary fiber: a review. Int J Biol Macromol. (2013) 61:1–6. doi: 10.1016/j.ijbiomac.2013.06.044, PMID: 23831534

[19] Drochner W. Digestion of carbohydrates in the pig. EAAP Public. (1991) 54:367–88. doi: 10.1080/17450399309386027 8390236

[20] Knudsen KEB Laerke HN Ingerslev AK Hedemann MS Nielsen TS Theil PK. Carbohydrates in pig nutrition - recent advances. J Anim Sci. (2016) 94:1–11. doi: 10.2527/jas.2015-978526812306

[21] Isaacson R Kim HB. The intestinal microbiome of the pig. Anim Health Res Rev. (2012) 13:100–9. doi: 10.1017/S1466252312000084 22853934

[22] Ma J Chen J Gan M Chen L Zhao Y Zhu Y. Gut microbiota composition and diversity in different commercial swine breeds in early and finishing growth stages. Animals (Basel). (2022) 12:1607. doi: 10.3390/ani12131607 35804507 PMC9264831

[23] Kim HB Isaacson RE. The pig gut microbial diversity: understanding the pig gut microbial ecology through the next generation high throughput sequencing. Vet Microbiol. (2015) 177:242–51. doi: 10.1016/j.vetmic.2015.03.014, PMID: 25843944

[24] Kim HB Borewicz K White BA Singer RS Sreevatsan S Tu ZJ. Longitudinal investigation of the age-related bacterial diversity in the feces of commercial pigs. Vet Microbiol. (2011) 153:124–33. doi: 10.1016/j.vetmic.2011.05.021 21658864

[25] Luo YH Ren W Smidt H Wright ADG Yu B Schyns G. Dynamic distribution of gut microbiota in pigs at different growth stages: composition and contribution. Microbiol Spectr. (2022) 10:e0068821. doi: 10.1128/spectrum.00688-21, PMID: 35583332 PMC9241710

[26] Holman DB Brunelle BW Trachsel J Allen HK. Meta-analysis to define a Core microbiota in the swine gut. Msystems. (2017) 2:e00004–17. doi: 10.1128/mSystems.00004-17, PMID: 28567446 PMC5443231

[27] Selvendran RR. The plant cell wall as a source of dietary fiber: chemistry and structure. Am J Clin Nutr. (1984) 39:320–37. doi: 10.1093/ajcn/39.2.320, PMID: 6320629

[28] Metzler BU Mosenthin R. A review of interactions between dietary fiber and the gastrointestinal microbiota and their consequences on intestinal phosphorus metabolism in growing pigs. Asian Austral J Anim. (2008) 21:603–15. doi: 10.5713/ajas.2008.r.03

[29] Thomas L Joseph A Gottumukkala LD. Xylanase and cellulase systems of clostridium sp.: an insight on molecular approaches for strain improvement. Bioresour Technol. (2014) 158:343–50. doi: 10.1016/j.biortech.2014.01.140, PMID: 24581864

[30] Krause DO Bunch RJ Smith WJM McSweeney CS. Diversity of Ruminococcus strains: a survey of genetic polymorphisms and plant digestibility. J Appl Microbiol. (1999) 86:487–95. doi: 10.1046/j.1365-2672.1999.00688.x

[31] Chassard C Delmas E Robert C Lawson PA Bernalier-Donadille A. *Ruminococcus champanellensis* sp nov., a cellulose-degrading bacterium from human gut microbiota. Int J Syst Evol Micr. (2012) 62:138–43. doi: 10.1099/ijs.0.027375-0 21357460

[32] Froidurot A Julliand V. Cellulolytic bacteria in the large intestine of mammals. Gut Microbes. (2022) 14:2031694. doi: 10.1080/19490976.2022.2031694, PMID: 35184689 PMC8865330

[33] Miron J Ben-Ghedalia D. Digestion of cell-wall monosaccharides of ryegrass and alfalfa hays by the ruminal bacteria Fibrobacter succinogenes and *Butyrivibrio fibrisolvens*. Can J Microbiol. (1993) 39:780–6. doi: 10.1139/m93-115 8221378

[34] Varel VH Fryda SJ Robinson IM. Cellulolytic bacteria from pig large intestine. Appl Environ Microbiol. (1984) 47:219–21. doi: 10.1128/aem.47.1.219-221.1984, PMID: 6696420 PMC239643

[35] Hespell RB. Microbial digestion of hemicelluloses in the rumen. Microbiol Sci. (1988) 5:362–5. 3079179

[36] Chassard C Delmas E Lawson PA Bernalier-Donadille A. *Bacteroides xylanisolvens* sp. nov., a xylan-degrading bacterium isolated from human faeces. Int J Syst Evol Microbiol. (2008) 58:1008–13. doi: 10.1099/ijs.0.65504-0 18398210

[37] Varel VH Robinson IM Jung HJ. Influence of dietary fiber on xylanolytic and cellulolytic bacteria of adult pigs. Appl Environ Microbiol. (1987) 53:22–6. doi: 10.1128/aem.53.1.22-26.1987, PMID: 3030194 PMC203595

[38] Martens EC Lowe EC Chiang H Pudlo NA Wu M McNulty NP. Recognition and degradation of plant Cell Wall polysaccharides by two human gut symbionts. PLoS Biol. (2011) 9:e1001221. doi: 10.1371/journal.pbio.1001221, PMID: 22205877 PMC3243724

[39] Flint HJ Scott KP Duncan SH Louis P Forano E. Microbial degradation of complex carbohydrates in the gut. Gut Microbes. (2012) 3:289–306. doi: 10.4161/gmic.19897, PMID: 22572875 PMC3463488

[40] Ze XL Duncan SH Louis P Flint HJ. *Ruminococcus bromii* is a keystone species for the degradation of resistant starch in the human colon. ISME J. (2012) 6:1535–43. doi: 10.1038/ismej.2012.4, PMID: 22343308 PMC3400402

[41] Reeves AR Wang GR Salyers AA. Characterization of four outer membrane proteins that play a role in utilization of starch by *Bacteroides thetaiotaomicron*. J Bacteriol. (1997) 179:643–9. doi: 10.1128/jb.179.3.643-649.1997, PMID: 9006015 PMC178742

[42] Gibson GR Beatty ER Wang X Cummings JH. Selective stimulation of bifidobacteria in the human colon by oligofructose and inulin. Gastroenterology. (1995) 108:975–82. doi: 10.1016/0016-5085(95)90192-2, PMID: 7698613

[43] Tanaka R Takayama H Morotomi M Kuroshima T Ueyama S Matsumoto K. Effects of administration of TOS and Bifidobacterium breve 4006 on the human Fecal Flora. Bifidobacteria Microflora. (1983) 2:17–24. doi: 10.12938/bifidus1982.2.1_17

[44] Barrangou R Azcarate-Peril MA Duong T Conners SB Kelly RM Klaenhammer TR. Global analysis of carbohydrate utilization by *Lactobacillus acidophilus* using cDNA microarrays. Proc Natl Acad Sci U S A. (2006) 103:3816–21. doi: 10.1073/pnas.0511287103, PMID: 16505367 PMC1533782

[45] Saulnier DAA Molenaar D de Vos WA Gibson GR Kolida S. Identification of prebiotic fructooligosaccharide metabolism in *Lactobacillus plantarum* WCFS1 through microarrays. Appl Environ Microb. (2007) 73:1753–65. doi: 10.1128/AEM.01151-06, PMID: 17261521 PMC1828832

[46] Poulsen HV Willink FW Ingvorsen K. Aerobic and anaerobic cellulase production by *Cellulomonas uda*. Arch Microbiol. (2016) 198:725–35. doi: 10.1007/s00203-016-1230-8, PMID: 27154570 PMC4995238

[47] Bayer EA Shimon LJ Shoham Y Lamed R. Cellulosomes-structure and ultrastructure. J Struct Biol. (1998) 124:221–34. doi: 10.1006/jsbi.1998.4065, PMID: 10049808

[48] Himmel ME Xu Q Luo Y Ding S-Y Lamed R Bayer EA. Microbial enzyme systems for biomass conversion: emerging paradigms. Biofuels. (2010) 1:323–41. doi: 10.4155/bfs.09.25

[49] Rincon MT Cepeljnik T Martin JC Barak Y Lamed R Bayer EA. A novel cell surface-anchored cellulose-binding protein encoded by the sca gene cluster of *Ruminococcus flavefaciens*. J Bacteriol. (2007) 189:4774–83. doi: 10.1128/JB.00143-07, PMID: 17468247 PMC1913464

[50] Morrison M Miron J. Adhesion to cellulose by *Ruminococcus albus*: a combination of cellulosomes and Pil-proteins? FEMS Microbiol Lett. (2000) 185:109–15. doi: 10.1111/j.1574-6968.2000.tb09047.x, PMID: 10754233

[51] Flint HJ Bayer EA Rincon MT Lamed R White BA. Polysaccharide utilization by gut bacteria: potential for new insights from genomic analysis. Nat Rev Microbiol. (2008) 6:121–31. doi: 10.1038/nrmicro1817 18180751

[52] Liu GS Li PH Hou LM Niu Q Pu G Wang BB. Metagenomic analysis reveals new microbiota related to Fiber digestion in pigs. Front Microbiol. (2021) 12:12. doi: 10.3389/fmicb.2021.746717 PMC8637618 34867862

[53] Grondin JM Tamura K Dejean G Abbott DW Brumer H. Polysaccharide Utilization Loci: Fueling Microbial Communities. J Bacteriol. (2017) 199:e00860-16. doi: 10.1128/JB.00860-16, PMID: 28138099 PMC5512228

[54] Terrapon N Lombard V Gilbert HJ Henrissat B. Automatic prediction of polysaccharide utilization loci in Bacteroidetes species. Bioinformatics. (2015) 31:647–55. doi: 10.1093/bioinformatics/btu716, PMID: 25355788

[55] Raut MP Karunakaran E Mukherjee J Biggs CA Wright PC. Influence of substrates on the surface characteristics and membrane proteome of *Fibrobacter succinogenes* S85. PLoS One. (2015) 10:e0141197. doi: 10.1371/journal.pone.0141197, PMID: 26492413 PMC4619616

[56] Jun HS Qi M Gong J Egbosimba EE Forsberg CW. Outer membrane proteins of *Fibrobacter succinogenes* with potential roles in adhesion to cellulose and in cellulose digestion. J Bacteriol. (2007) 189:6806–15. doi: 10.1128/JB.00560-07 17644604 PMC2045214

[57] Birt DF Boylston T Hendrich S Jane JL Hollis J Li L. Resistant starch: promise for improving human health. Adv Nutr. (2013) 4:587–601. doi: 10.3945/an.113.004325, PMID: 24228189 PMC3823506

[58] Gutierrez TJ Tovar J. Update of the concept of type 5 resistant starch (RS5): self-assembled starch V-type complexes. Trends Food Sci Tech. (2021) 109:711–24. doi: 10.1016/j.tifs.2021.01.078

[59] Giuberti G Gallo A Moschini M Masoero F. New insight into the role of resistant starch in pig nutrition. Anim Feed Sci Tech. (2015) 201:1–13. doi: 10.1016/j.anifeedsci.2015.01.004

[60] Macfarlane GT Englyst HN. Starch utilization by the human large intestinal microflora. J Appl Bacteriol. (1986) 60:195–201. doi: 10.1111/j.1365-2672.1986.tb01073.x, PMID: 2423494

[61] Reeves AR D'Elia JN Frias J Salyers AA. A *Bacteroides thetaiotaomicron* outer membrane protein that is essential for utilization of maltooligosaccharides and starch. J Bacteriol. (1996) 178:823–30. doi: 10.1128/jb.178.3.823-830.1996, PMID: 8550519 PMC177731

[62] Ze XL Ben David Y Laverde-Gomez JA Dassa B Sheridan PO Duncan SH. Unique Organization of Extracellular Amylases into Amylosomes in the resistant starch-utilizing human colonic firmicutes bacterium *Ruminococcus bromii*. MBio. (2015) 6:e01058–15. doi: 10.1128/mBio.01058-15, PMID: 26419877 PMC4611034

[63] Cockburn DW Orlovsky NI Foley MH Kwiatkowski KJ Bahr CM Maynard M. Molecular details of a starch utilization pathway in the human gut symbiont *Eubacterium rectale*. Mol Microbiol. (2015) 95:209–30. doi: 10.1111/mmi.12859, PMID: 25388295 PMC4437465

[64] Ramsay AG Scott KP Martin JC Rincon MT Flint HJ. Cell-associated alpha-amylases of butyrate-producing firmicute bacteria from the human colon. Microbiology (Reading). (2006) 152:3281–90. doi: 10.1099/mic.0.29233-0 17074899

[65] Englyst KN Liu S Englyst HN. Nutritional characterization and measurement of dietary carbohydrates. Eur J Clin Nutr. (2007) 61:S19–39. doi: 10.1038/sj.ejcn.1602937 17992185

[66] Walter J. Ecological role of lactobacilli in the gastrointestinal tract: implications for fundamental and biomedical research. Appl Environ Microb. (2008) 74:4985–96. doi: 10.1128/AEM.00753-08, PMID: 18539818 PMC2519286

[67] Sako T Matsumoto K Tanaka R. Recent progress on research and applications of non-digestible galacto-oligosaccharides. Int Dairy J. (1999) 9:69–80. doi: 10.1016/S0958-6946(99)00046-1

[68] Louis P Flint HJ. Diversity, metabolism and microbial ecology of butyrate-producing bacteria from the human large intestine. FEMS Microbiol Lett. (2009) 294:1–8. doi: 10.1111/j.1574-6968.2009.01514.x, PMID: 19222573

[69] Miller TL Wolin MJ. Pathways of acetate, propionate, and butyrate formation by the human fecal microbial flora. Appl Environ Microbiol. (1996) 62:1589–92. doi: 10.1128/aem.62.5.1589-1592.1996, PMID: 8633856 PMC167932

[70] Silva YP Bernardi A Frozza RL. The role of short-chain fatty acids from gut microbiota in gut-brain communication. Front Endocrinol. (2020) 11:11. doi: 10.3389/fendo.2020.00025 PMC7005631 32082260

[71] Topping DL Clifton PM. Short-chain fatty acids and human colonic function: roles of resistant starch and nonstarch polysaccharides. Physiol Rev. (2001) 81:1031–64. doi: 10.1152/physrev.2001.81.3.1031, PMID: 11427691

[72] Gardiner GE Metzler-Zebeli BU Lawlor PG. Impact of intestinal microbiota on growth and feed efficiency in pigs. Review Microorganisms. (2020) 8:1886. doi: 10.3390/microorganisms8121886 33260665 PMC7761281

[73] Duncan SH Holtrop G Lobley GE Calder AG Stewart CS Flint HJ. Contribution of acetate to butyrate formation by human faecal bacteria. Br J Nutr. (2004) 91:915–23. doi: 10.1079/BJN20041150 15182395

[74] De Vadder F Kovatcheva-Datchary P Goncalves D Vinera J Zitoun C Duchampt A. Microbiota-generated metabolites promote metabolic benefits via gut-brain neural circuits. Cells. (2014) 156:84–96. doi: 10.1016/j.cell.2013.12.016, PMID: 24412651

[75] Nogal A Louca P Zhang X Wells PM Steves CJ Spector TD. Circulating levels of the short-chain fatty acid acetate mediate the effect of the gut microbiome on visceral fat. Front Microbiol. (2021) 12:711359. doi: 10.3389/fmicb.2021.711359, PMID: 34335546 PMC8320334

[76] Blad CC Tang C Offermanns S. G protein-coupled receptors for energy metabolites as new therapeutic targets. Nat Rev Drug Discov. (2012) 11:603–19. doi: 10.1038/nrd3777, PMID: 22790105

